# Worsening of pretibial myxedema following radioiodine treatment for hyperthyroid Graves’ disease

**DOI:** 10.11604/pamj.2023.44.85.36177

**Published:** 2023-02-14

**Authors:** Yotsapon Thewjitcharoen, Thep Himathongkam

**Affiliations:** 1 Diabetes and Thyroid Center, Theptarin Hospital, Bangkok, Thailand

**Keywords:** Pretibial myxedema, radioiodine, worsening

## Image in medicine

The development of pretibial myxedema following RAI treatment has been occasionally reported from the probable influences of thyrotropin receptor antibodies (TRAbs) similar to the RAI-associated GO. Herein, we report an interesting case of the fluctuating clinical course of pretibial myxedema following treatments of hyperthyroid Graves’ disease for over 3 years. A 49-year-old Thai woman with underlying hyperthyroid Graves’ disease with Graves’ orbitopathy (GO) and pretibial myxedema for 1 year came to our hospital for relapsed hyperthyroidism and active GO. A plague form of pretibial myxedema with indurated hyperpigmented lesions was found on both legs (A). Intravenous pulse methylprednisolone (IVMP) was given to control her active GO. Both GO and pretibial myxedema significantly improved after IVMP treatments (B). Then, radioiodine (RAI) treatment (30 mCi) was administered at 1 month later. Transient hypothyroidism developed at 4 months post-RAI and the estimated weights of the thyroid decreased from 60 grams to 30 grams. However, hyperthyroidism relapsed again within 6 months post-RAI treatment. Eventually, a second dose of RAI (20 mCi) was given. At that time, her pretibial myxedema flared-up (C) but her GO was still inactive. Topical corticosteroid ointment was given for pretibial myxedema and only local supportive measures were applied to treat GO. Finally, permanent hypothyroidism was achieved at 6 months after the second course of RAI. Currently, her pretibial myxedema is stable without further flare-up (D). Our case highlights the potential of RAI treatment to precipitate pretibial myxedema in patients with pre-existing skin lesions.

**Figure 1:**
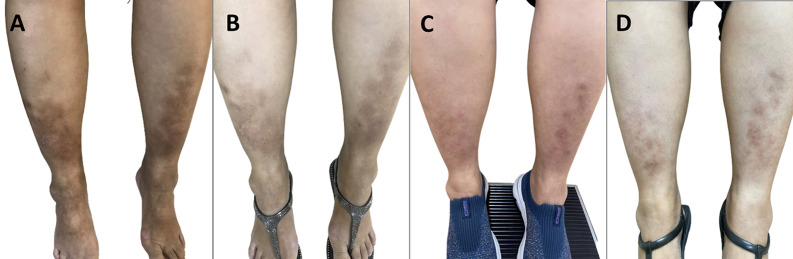
clinical evolution of pretibial myxedema in this patient over a period of 3 years; A) typical appearance of plague-type pretibial myxedema at the first visit in our hospital; B) partial remission of pretibial myxedema following intravenous pulse methylprednisolone for Graves’ ophthalmopathy (1 month after the first visit); C) worsening of pretibial myxedema following the second dose of RAI treatment for 4 months (28 months after the first visit); D) stable pretibial myxedema at the last visit with permanent hypothyroidism (36 months after the first visit)

